# First Report of Root-Knot Nematode, *Meloidogyne incognita*, on Tree Houseleek (*Aeonium arboreum*) in the United States

**DOI:** 10.2478/jofnem-2025-0031

**Published:** 2025-08-31

**Authors:** Anil Baniya, Omar Zayed, Perla Achi, Pierluigi Perfetto, Adler Dillman

**Affiliations:** Department of Nematology, University of California, Riverside, 900 University Ave., Riverside, CA 92521; Department of Botany and Plant Science, University of California, Riverside, 900 University Ave., Riverside, CA 92521; Department of Soil, Plant and Food Science, University of Bari, Via Amendola 165/A, 70126 Bari, Italy

**Keywords:** Diagnosis, California, *Aeonium*, mitochondrial haplotyping, root galls, root-knot nematode, species-specific primers

## Abstract

*Aeonium*, or tree houseleek (*Aeonium arboreum*), is a bushy, perennial succulent and a popular ornamental plant in regions such as California, New Zealand, Australia, Sicily, Gibraltar, and Chile. It features rosettes of soft, waxy leaves at the tips of sparsely branched or occasionally single, bare stems. It is drought-tolerant and has a variety of colors and forms, making it a popular ornamental plant. In July 2024, a diseased *Aeonium* plant was submitted by a home gardener from Los Angeles County, California, to the Department of Nematology at the University of California, Riverside (UCR), for diagnosis. Root galls were observed on the plant, and further examination revealed high numbers of root-knot nematodes (*Meloidogyne* sp.). Molecular species identification was conducted using ribosomal DNA, mitochondrial haplotyping, and species-specific primer techniques, including the TRNAH/MHR106 and MORF/MTHIS primer sets, along with *Meloidogyne incognita*-specific primers (MIF/MIR). Amplification and sequencing of the marker genes identified the root-knot nematode infecting *Aeonium* as *M. incognita*. To our knowledge, this study presents the first report of *M. incognita* infecting *Aeonium* worldwide.

## Introduction

The genus *Aeonium* includes succulent plants belonging to the Crassulaceae family, comprising approximately 35 species native to northern Africa and the Canary Islands. *Aeonium arboreum* is a bushy, perennial succulent characterized by rosettes of soft, waxy leaves at the tips of sparsely branched or sometimes single, bare stems. When mature, the rosettes produce yellowish flower clusters. *Aeoniums* are commonly grown as ornamental plants in gardens and containers (Rizza et al., 2011). *Aeoniums* have also become naturalized and established in various regions around the world, including several coastal areas of New Zealand, Australia, Sicily, Gibraltar, Chile, and California (Schulz, 2007).

Tree houseleek (*Aeonium arboreum*) is thought to have been introduced to California in the early 20th century (San Marcos Growers, 2024). The name *Aeonium* comes from the Greek word ‘aionion,’ meaning ‘everlasting plant,’ reflecting the plant’s succulent characteristics and presumed long lifespan (Walker, 2021). This plant thrives in full to partial sun in coastal regions and prefers partial shade when grown inland. It requires occasional to infrequent watering, exhibiting drought tolerance in coastal gardens. It can endure temperatures between 4 and 38°C, making it well-suited for coastal environments, and is also resistant to deer feeding. Ideal for California’s winter rain patterns, it comes in a variety of forms, ranging from ground-hugging to shrubby, with a diverse range of colors and textures, including green, red, and plum, as well as smooth, fuzzy, or serrated leaves. This versatility allows multiple varieties to fit seamlessly into almost any garden without appearing redundant (Schulz, 2007). These characteristics make *Aeonium* a popular horticultural choice in California, where it can be found in a variety of shapes and forms in home gardens, botanical and desert gardens, and natural habitats. It is also widely available at nurseries. California’s climate, characterized by mild, wet winters and hot, dry summers, similar to that of the Mediterranean region, provides an ideal environment for the tree houseleek (Swain, 2021). Typically, the region’s vegetation begins to dry out in late spring or early summer as soil moisture diminishes, reaching its maximum dryness by late summer or early autumn. While most grasses die off due to dry conditions by late spring (Parker and Schimel, 2011), the drought-resistant tree houseleek can survive, potentially serving as a reservoir for pathogens. In contrast, in the Midwestern United States, *Aeoniums* cannot survive the winter outdoors, but they are commonly grown in containers (Mahr, 2024).

Although *Aeonium* is a well-known ornamental plant, there is limited information on its pathogens and diseases. In 1973, *Aeonium* sp. Webb & Berth was reported as a susceptible host to the cyst nematode (*Heterodera* sp.) in California (Siddiqui et al., 1973). Additionally, infection by Tobacco ringspot virus (TRSV) has been recorded in *Aeonium* spp. in Southern Italy, where affected plants exhibited chlorotic spots and rings on both leaf surfaces (Sorrentino et al., 2013). TRSV is vectored by the nematode *Xiphinema americanum* (McGuire, 1973), making this one of only two reported instances of potential plant-parasitic nematode associations with *Aeonium*. In addition, stem blight has been observed in *Aeonium* species in Egypt (Abdel-Kader et al., 2010).

Root-knot nematodes (*Meloidogyne* spp.) are highly polyphagous plant pathogens due to their remarkably broad host range, which includes approximately 250,000 flowering plant species (Trudgill and Blok, 2001). However, there is limited or no information available regarding their ability to infect *Aeonium* species. In July 2024, an *Aeonium arboretum* plant displaying symptoms of root disease was submitted to the Department of Nematology at the University of California, Riverside (UCR) for diagnosis, originating from Los Angeles. The roots showed extensive galling (Figure 2A and B), with female root-knot nematodes observed within the galls. Further examination revealed a high presence of root-knot nematodes (*Meloidogyne* sp.) in the roots. Since there have been no previous reports of root-knot nematode infections in *Aeonium*, a study was conducted to identify the nematode species extracted from *Aeonium* roots using mitochondrial haplotyping and species-specific primers. The study also aimed to confirm whether *Aeonium* is a host for root-knot nematodes.

**Figure 1:**
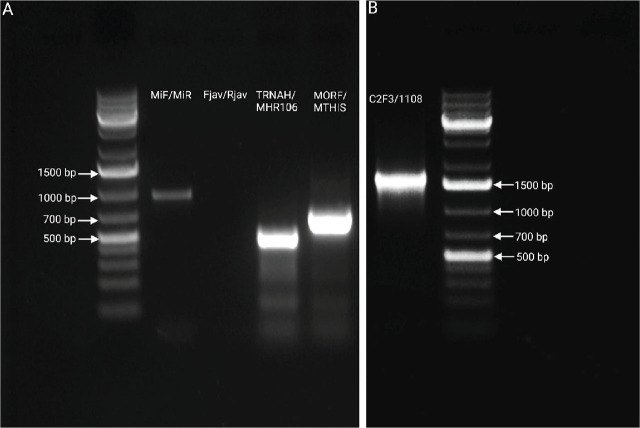
(A) Amplified products were obtained using *M. incognita*-specific (MIF/MIR) and *M. javanica*-specific (Fjav/Rjav) primers. Only the MIF/MIR primers produced a 999-bp band, indicating that the root-knot nematode infecting the *Aeonium* was *M. incognita*, while no amplification was observed with the Fjav/Rjav primers. Two mitochondrial DNA regions, covering the intergenic spacer and part of the adjacent large subunit ribosomal RNA gene (lrDNA), were amplified using the TRNAH/MHR106 (approximately 550-bp) and MORF/MTHIS (approximately 750 bp) primer sets; (B) The intergenic spacer, tRNA^His^, and the large subunit ribosomal RNA gene (rRNA) were amplified using the C2F3/1108 primers, yielding an approximately 1550-bp product.

**Figure 2:**
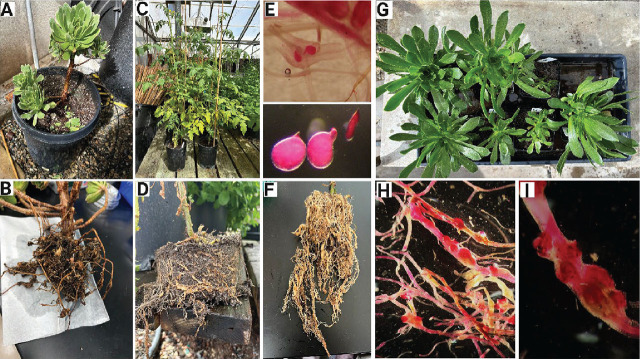
(A) Tree houseleek (*Aeonium arboreum*) plant received from Los Angeles, California for diagnosis; (B) Heavy galling on the plant roots, indicating root-knot nematode (RKN) infection; (C) Tomato plants used to test for RKN infection; (D) and (F) Visible galls on the infected tomato roots, both in the pot and after washing; (E) Acid fuchsin staining of the tomato roots, showing the female nematodes inside the roots, along with the morphology of adult and early female nematodes; (G) Cuttings of the *Aeonium* plants inoculated with nematodes; (H and I) Acid fuchsin staining of the *Aeonium arboreum* cuttings, showing characteristic symptoms of nematode infection.

## Materials and Methods

### Nematode population and extraction

In July 2024, a diseased *Aeonium* plant was submitted to the Department of Nematology at UCR for diagnosis by a Los Angeles County, California home gardener. The plant had been grown in a pot for its entire life. Roots of the diseased plant were washed gently with tap water to remove adhering soil particles, and the cleaned roots were used to extract plant-parasitic nematode eggs. The roots were cut into small pieces and placed in a jar containing 1% bleach. The eggs were extracted and hatched. Briefly, a clean metal basket was lined with Kimwipes and placed on a glass Petri dish, leaving a small gap between the basket bottom and the dish. Nematode eggs were added to the paper, and enough tap water was added so the basket just touched the water surface without submerging; the setup was covered with aluminum foil. J2s were collected from the dish after 4 days, following the procedure outlined by Atamian et al. (2012). The hatched juveniles were then inoculated and mass-produced in a susceptible cultivar of tomato (Moneymaker). A month after inoculation, eggs were extracted from the infected roots. The juveniles were hatched as previously described and used to inoculate plants generated from cuttings of *Aeonium arboreum*.

### Molecular species identification of a root-knot nematode

Approximately 20 freshly emerged juveniles were used for DNA extraction. The juveniles were pipetted into a 0.2 ml microcentrifuge polymerase chain reaction (PCR) tube mixed with 18 μl of 10 mM Tris, 1 mM EDTA, 1 μl of 2% Triton X, and 1 μl of Proteinase K (20 mg/ml, New England Biolabs, Ipswich, Massachusetts). The nematodes’ cuticle was disrupted by subjecting the sample to three cycles of freezing and thawing with liquid nitrogen, followed by overnight incubation of the nematode at −20°C. The next day, the frozen lysate was incubated at 56°C for 1 hour, then heated to 95°C for 10 minutes to extract DNA. PCR reaction was performed with a total volume of 25 μl, which included 2 μl of genomic DNA as the template, 12.5 μl of 2X Taq Red Master Mix kit (Genesee Scientific, El Cajon, CA, USA), 1.25 μl each of forward and reverse primers at a concentration of 10 μM, and 8 μl of nuclease-free water. We first amplified the ribosomal DNA (rDNA) regions to identify the genus of the nematode. For ITS rDNA, we performed PCR amplification using the primers TW81 (5′-GTTTCCGTAGGTGAACCTGC-3′) and AB28 (5′-ATATGCTTAAGTTCAGCGGGT-3′) (Joyce et al., 1994). For 18S (5′-TTGATTACGTCCCTGCCCTTT-3′) and 26R (5′-TTTCACTCGCCGTTACTAAGG-3′) (Vrain et al. 1992). Similarly, the 28S rDNA gene was amplified using D2F (5′-CCTTAGTAACGGCGAGTGAAA-3′) and 536 (5′-CAGCTATCCTGAGGAAAC-3′) primers (Nguyen et al., 2006). Identical conditions were employed for the PCR amplification of three loci: an initial denaturation step at 96°C for 5 minutes, followed by 35 cycles of denaturation at 96°C for 1 minute, annealing at 57°C for 45 seconds, and extension at 72°C for 1 minute and a final extension at 72°C for 10 minutes.

To achieve species-level identification of the root-knot nematode, we employed mitochondrial haplotyping along with species-specific primers. Genomic DNA was extracted from four female nematodes collected from infected tomato roots, following the method previously described. Two mitochondrial DNA regions spanning the intergenic spacer and part of the adjacent large subunit ribosomal RNA gene (lrDNA) were amplified. The primer set TRNAH (5′-TGAATTTTTTATTGTGATTAA-3′) and MRH106 (5′-AATTTCTAAAGACTTTTCTTAGT-3′) targeted the tRNA^His^ and lrDNA regions, while the MORF (5′-ATCGGGGTTTAATAATGGG-3′) and MTHIS (5′-AAATTCAATTGAAATTAATAGC-3′) primers amplified the intergenic spacer and tRNA^His^ region (Pagan et al., 2015; Stanton et al., 1997). The thermocycling conditions for amplifying both genes were applied according to (Pagan et al., (2015). Once the mitochondrial haplotype was identified, *Meloidogyne incognita*-specific MIF: 5′-GTGAGGATTCAGCTCCCCAG-3′ and MIR: 5′-ACGAGGAACATACTTCTCCGTCC-3′ and *M. javanica*-specific Fjav (5′-GGTGCGCGATTGAACTGAGC-3′) and Rjav (5′-CAGGCCCTTCAGTGGAACTATAC-3′) primers were employed to confirm the species of the root-knot nematode (Adam et al., 2007).

Upon confirmation of species, the mitochondrial region encompassing the cytochrome oxidase II (COII) gene, the intergenic spacer, tRNA^His^, and the large subunit ribosomal RNA (lrRNA) gene was amplified using the primers C2F3 (5′-GGTCAATGTTCAGAAATTTGTGG-3′) and 1108 (5′-TACCTTTGACCAATCACGCT-3′) (Powers et al., 2005; Powers and Harris, 1993). Thermocycling conditions were similar to those above except for the annealing time of 90 seconds. All the PCR products were analyzed using a 1% agarose gel stained with 0.0003% ethidium bromide and a 1-kb plus DNA ladder (New England Biolabs). The PCR products were then purified using the QIAquick® PCR Purification Kit (Qiagen, Germantown, Maryland), according to the manufacturer’s protocol. The purified products were sequenced in both forward and reverse directions using Sanger sequencing at the UCR Core Instrumentation Facility. Forward and reverse sequences of each gene were aligned to obtain consensus sequences using SeqManII software (DNASTAR, Madison, Wisconsin). Any ambiguities in the chromatogram sequences of both strands for each locus were visually inspected and manually corrected. The consensus sequences generated from all sequencing were compared with those available in the National Center for Biotechnology Information (NCBI) GenBank database using the Basic Local Alignment Search Tool (BLAST) to assess sequence homology.

### Evaluation of root-knot nematode infection on *Aeonium arboreum*

Young plants of *Aeonium arboreum* were propagated from the mother plant using leaf cuttings and stem cuttings. Healthy, undamaged leaves and branches were carefully collected from the mother plant. Stems were cut into small sections about 4 inches long, dipped in auxin at the base, and planted in individual pots filled with UC soil mix III (a mixture of plaster sand, peat moss, KNO_3_, limestone flour, phosphate, dolomite, magnesium, iron, manganese, zinc, and copper). Individual healthy leaves were also placed in separate pots with the same soil mixture. The cuttings were allowed to grow for 45 days, after which 1,000 freshly hatched root-knot nematode juveniles were inoculated to each cutting (n=5). The nematode-inoculated plants were allowed to grow for one month. At harvest, the root systems were removed from the pots, rinsed with tap water, and examined for root-knot nematode infection. Nematodes within the roots were stained with acid fuchsin (Bybd et al., 1983). After staining, the infected roots were rated for galling and egg masses on a scale from 0 to 5, where 0 = 0 galls or egg masses, 1 = 1–2 galls or egg masses, 2 = 3–10 galls or egg masses, 3 = 11–30 galls or egg masses, 4 = 31–100 galls or egg masses, and 5 = ≥100 galls or egg masses per root (Sasser et al., 1984).

## Results and Discussion

Upon receiving an *Aeonium* plant showing symptoms of nematode infection, we initially identified the nematode using ribosomal DNA. Amplification with the ribosomal DNA using TW81/AB28 primers produced a 409 bp fragment, the 18S/26R primers yielded a 708 bp fragment, and the D2F/536 primer generated a 1011 bp fragment. When compared with available sequences at NCBI, all these fragments were 96 %, 99 %, and 99 % identical, respectively, to the MT490922, MT209948, and MK292132 sequences of *Meloidogyne incognita*. This rDNA offers important insights and has helped with species identification; however, there is evidence of sequence variation in the genes among specific populations of root-knot nematodes. Importantly, the conserved ribosomal DNA cannot distinguish between the different species of tropical RKN, including *M. incognita*, as the variation within individual RKNs is greater than that observed between species (Bogale et al., 2020; Gissi et al., 2008; Pagan et al., 2015; Ye et al., 2015).

Root-knot nematodes (*Meloidogyne incognita*) are mitotic parthenogens (apomicts), which reproduce asexually (Janssen et al., 2017). Because of its uniparental inheritance, the mitochondrial genome can be used as a diagnostic marker that can resolve some of the challenges associated with distinguishing these species (Powers and Harris 1993; Stanton et al. 1997; Powers et al. 2005; Gissi et al. 2008; Adams et al. 2009). A diagnostic tool was developed to assign mitochondrial haplotypes based on the size of the intergenic spacers and sequence variations in the large subunit (16S) rRNA (lrDNA). This tool amplifies two mitochondrial regions that together encompass the intergenic spacer and a portion of the adjacent lrDNA (Pagan et al., 2015). Genomic DNA amplified from four randomly selected females in the present study showed that the sizes of the amplified products were approximately 742 bp for the MORF/MTHIS primer sets and 557 bp for the TRNAH/MRH106 primers. However, this mitochondrial haplotype size could be associated with either *M. javanica* or *M. incognita*. To distinguish between the two species, we utilized two species-specific SCAR (sequence characterized amplified region) markers: MIF/MIR for *M. incognita* and Fjav/Rjav for *M. javanica* (Adam et al. 2007). Amplification with MIF/MIR primers produced an approximately 999-bp PCR product, whereas PCR using Fjav/Rjav primers resulted in no amplification of the genomic DNA (Figure 1).

All amplified DNA fragments from our samples were sequenced and compared with available sequences in the NCBI database. The fragment amplified using the TRNAH/MRH106 primer set was 99 % identical (100 % query cover), and the fragment from the MORF/MTHIS primer set was 99 % identical (100 % query cover) to the OM523730 and OM523797 sequences of *Meloidogyne incognita*, respectively. Similarly, the sequence from the amplified product of the MIF/MIR primer set was 100% identical (99% query cover) to *M. incognita* with accession numbers JN005841 and OQ919497. This confirms that the root-knot nematode infecting the *Aeonium* roots was *M. incognita*. The amplified product using C2F3/1108 primers yielded an approximately 1127-bp product, and the sequence showed 99% identity (100% query cover) to multiple *M. incognita* sequences in the NCBI database, including JX100437 and FJ159624. The newly acquired consensus sequences from this study are deposited in the NCBI GenBank database under the following accession numbers: ribosomal genes PQ999814, PQ999813, PV013884, and mitochondrial genes PV151353, PV151352, PV008658, PV151351.

To verify Koch’s postulates, we inoculated 10 tomato plants (Moneymaker) with approximately 2,000 J2 juveniles hatched from eggs extracted from *Aeonium* roots. Approximately 45 days after infection, the plants were removed from the pots, and the soil was gently removed from the roots. Many galls and egg masses were found on each plant’s roots, and second-stage juveniles (J2) and females were isolated. These eggs were then hatched again and inoculated onto plants grown from *Aeonium* cuttings.

The staining of root cuttings showed an average of 47 female nematodes or egg masses on the plant roots. According to the matrices developed by Sasser et al. (1984), the houseleek has a gall index (GI) or egg mass index (EI) of 4, corresponding to 31–100 galls or egg masses per plant. This indicates that *Aeonium* is a susceptible host for *Meloidogyne incognita*. Based on our research findings, we recommend that all commercial nurseries, home gardeners, and private sellers of *Aeonium* consider the plant’s susceptibility to *M. incognita,* avoid using RKN-contaminated soil, and follow all phytosanitary measures to maintain healthy plants. To the best of our knowledge, this is the first documented case of *M. incognita* infecting *Aeonium* globally.
